# Long-Term Effectiveness of Secukinumab in Patients with Axial Spondyloarthritis

**DOI:** 10.1155/2020/6983272

**Published:** 2020-03-31

**Authors:** Stefano Gentileschi, Donato Rigante, Jurgen Sota, Giuseppe Lopalco, Maria Grazia Giannotta, Giacomo Emmi, Gerardo Di Scala, Florenzo Iannone, Claudia Fabiani, Bruno Frediani, Luca Cantarini

**Affiliations:** ^1^Research Center of Systemic Autoinflammatory Diseases and Behçet's Disease and Rheumatology-Ophthalmology Collaborative Uveitis Center, Department of Medical Sciences, Surgery and Neurosciences, University of Siena, Siena, Italy; ^2^Institute of Pediatrics, Fondazione Policlinico Universitario A. Gemelli IRCCS, Rome, Italy; ^3^Università Cattolica del Sacro Cuore, Rome, Italy; ^4^Department of Emergency and Organ Transplantation, Rheumatology Unit, University of Bari, Bari, Italy; ^5^Department of Experimental and Clinical Medicine, University of Florence, Florence, Italy; ^6^Ophthalmology Unit, Department of Medicine, Surgery and Neuroscience, University of Siena, Siena, Italy

## Abstract

**Objectives:**

The primary aim of our study was to evaluate long-term efficacy of secukinumab (SCK) in patients with axial spondyloarthritis (axSpA); secondary aims were to evaluate drug retention rate and to identify differences in the clinical and laboratory assessment according to axSpA clinical features, dosage administered, and biologic treatment lines. *Patients and Methods*. We collected clinical, demographical, and treatment data from 39 patients affected by axSpA consecutively treated with SCK. Laboratory assessment was based on inflammation parameters; clinical assessment was performed with the Ankylosing Spondylitis Disease Activity Score- (ASDAS-) CRP and Bath Ankylosing Spondylitis Disease Activity Index (BASDAI). Data were recorded at baseline and every 3 months for the first year and then every 6 months in the second year.

**Results:**

Twelve males and 27 females were enrolled; both BASDAI and ASDAS-CRP showed a statistically significant reduction during the observation period (*p* < 0.0001 and *p* < 0.0001, respectively). C-reactive protein significantly decreased (*p* = 0.006), with significant reduction at the post hoc analysis between baseline and both 6-month evaluation (*p* = 0.02) and 24-month visit (*p* = 0.036). No statistical significance was observed in BASDAI and ASDAS-CRP improvement (*p* = 0.482 and *p* = 0.164, respectively) between different dosages administered. No significant differences emerged in the BASDAI and ASDAS-CRP variations between biologic-naïve patients and subjects previously failing to tumour necrosis factor (TNF) inhibitors (*p* = 0.53 and *p* = 0.148, respectively). At the end of our observation, 7 out of 39 patients discontinued SCK. The global retention rate at the end of the study period was 78.2%, without any significant differences between biologic-naïve and anti-TNF-failure patients (*p* = 0.619) or between subjects administered with different SCK dosages (*p* = 0.614). No adverse events were reported.

**Conclusions:**

In our cohort, SCK has proved a remarkable effectiveness regardless biologic treatment line and dosages employed. As suggested by the notable drug retention rate, SCK has been able to maintain its effectiveness over a considerable long period of treatment.

## 1. Introduction

Spondyloarthritis (SpA) is a group of chronic rheumatic diseases sharing genetic, clinical, and imaging features. SpA, which mainly occurs with axial symptoms, i.e., chronic low-back pain and stiffness tending to improve with exercise, is classified as axial SpA (axSpA) [1, 2]. Inflammatory bowel diseases (IBD), anterior uveitis, and psoriasis are some of the extra-articular manifestations often associated with SpA [3]. Until the more recent introduction of magnetic resonance imaging (MRI) of the sacroiliac joints, diagnosis of axSpA was based exclusively on radiographic findings, often leading to delays in starting treatment [4, 5]. MRI's ability of detecting early signs of sacroiliac joint inflammation led to a distinction between radiographic and nonradiographic axSpA, respectively, characterized by the presence or absence of suggestive findings at the conventional radiology [6]. Concerning treatment, nonsteroidal anti-inflammatory drugs (NSAIDs) still have a relevant role in managing axSpA manifestations, but their long-term use can often lead to the development of various side effects [7]. The over-time management and prognosis of axSpA radically changed over the last two decades since the introduction of tumour necrosis factor- (TNF-) *α* inhibitors [8–13]. However, it is known that up to 40% of patients do not benefit from TNF-*α* blockade due to loss of efficacy or drug tolerance issues [14–16]. Therefore, the need to find alternative treatments for these patients has led to the development of further drugs able to block another pivotal cytokine involved in axSpA inflammation, such as interleukin- (IL-) 17 [17]. The human anti-IL-17A monoclonal antibody secukinumab (SCK) has been approved for the treatment of ankylosing spondylitis (AS), after proving its effectiveness in 5 multicentre phase III trials, including 4 randomized double blind trials and their extensions (MEASURE 1 [18], MEASURE 2 [18], MEASURE 3 [19], MEASURE 4 [20], and MEASURE 2-J [21]).

The good clinical results described above are also confirmed by some real-life observational studies, including our experience which focused on the short-term clinical response and on the overall satisfaction related to treatment with SCK in patients with axSpA treated with different dosages and in various lines of biologic therapy (LoBT) [22–25]. We herein report our experience in a multicentre cohort of axSpA patients treated with SCK over a 24-month period.

## 2. Patients and Methods

We enrolled 39 patients diagnosed with axSpA and consecutively undergoing SCK treatment in three Italian referral centres (Siena, Bari, and Florence). Diagnosis of axSpA was performed according to the Assessment in SpondyloArthritis international Society (ASAS) criteria [26]. We excluded from the study any patient undergoing SCK for less than 12 months.

Every patient in the cohort underwent an induction scheme at the start of treatment, consisting in subcutaneous SCK 150 mg weekly for the first five injections, and then a maintaining dose of 150 mg every 4 weeks. In subjects affected by psoriasis, the dosage employed was 300 mg per administration.

Patients were treated in different LoBT, since some of the subjects were naïve to any biologic agent and others underwent SCK after lack or loss of response to previous anti-TNF-*α* agents. Before starting treatment, a full serological and instrumental screening was performed to rule out active infectious diseases. Patients presenting latent tuberculosis underwent a complete 6-month prophylaxis with isoniazid (400 mg/day).

Laboratory and clinical evaluation was performed at baseline and every 3 months for the first year. In the second year of treatment, follow-up visits were performed every 6 months. Laboratory assessment included C-reactive protein (CRP) and erythrocyte sedimentation rate (ESR). Disease activity was assessed using the Bath Ankylosing Spondylitis Disease Activity Index (BASDAI) and the Ankylosing Spondylitis Disease Activity Score- (ASDAS-) CRP.

The primary aim of the study was to evaluate SCK long-term efficacy in the management of axSpA manifestations. Secondary aims were as follows: (i) to assess drug survival; (ii) to identify differences in the clinical and laboratory assessment and drug survival according to the dosage administered or LoBT; (iii) to point out differences in the clinical assessment and drug survival according to different clinical features such as the presence of radiographic axSpA or psoriasis; and (iv) to report any adverse events eventually occurring in the cohort.

The primary endpoints were represented by the following: (i) a statistically significant reduction of ASDAS-CRP and BASDAI indexes over the period of observation and (ii) a statistically significant reduction of inflammatory markers (CRP and ESR) over the period of observation. Secondary endpoints were represented by the following: (i) evaluating drug retention rate; (ii) a statistically significant difference in drug retention rate and in the reduction of clinimetric parameters and inflammatory markers between patients undergoing SCK 150 mg and those treated with SCK 300 mg; (iii) a statistically significant difference in drug retention rate and in the reduction of clinimetric parameters and inflammatory markers between biologic-naïve patients and subjects previously treated with TNF-*α* inhibitors; (iv) a statistically significant difference in drug retention rate and in the reduction of clinimetric parameters between psoriatic and non-psoriatic patients and between radiographic and non-radiographic axSpA; and (v) the frequency of adverse events occurring during the treatment. The study was approved by the local Ethical Committee of the Azienda Ospedaliera Universitaria in Siena, Italy.

Data were computed using IBM SPSS Statistics for Windows, version 24 (IBM Corp., Armonk, NY, USA). Descriptive statistics was employed to calculate percentages, and data regarding continuous variables were summarized as means and standard deviations or medians and interquartile ranges as appropriate. The Shapiro-Wilk test was employed to evaluate the normal behavior of our sample. Means on multiple samples were compared with the Kruskal-Wallis test and Mann–Whitney *U* test for post hoc analysis or repeated measure ANOVA. The results were then corrected with the Bonferroni method. Means or medians on different samples were evaluated by an independent *t*-test or the Mann–Whitney *U* test as requested. Time-to-event analysis was performed according to the Kaplan-Meier method, and survival curves were compared with the log-rank test. The threshold for statistical significance was set at *p* < 0.05, and all *p* values were two-sided.

## 3. Results

Thirty-nine consecutive patients (12 males; 27 females) affected with axSpA and treated with SCK were enrolled in the study. Treatment duration (mean ± SD) was 19.04 ± 4.5 months. Clinical, demographic, and therapeutic data of the cohort are summarized in Table 1.

At the end of the observation period, 7 out of 39 (17.94%) patients discontinued SCK due to lack of efficacy (*n* = 2), loss of efficacy (*n* = 3), or poor compliance (*n* = 2).

Ten patients (25.64%) were administered with SCK as a first-line biologic agent, while the other 29 subjects (74.35%) had previously experienced a failure to one (*n* = 8, 27.58%), two (*n* = 13, 44.82%), three (*n* = 6, 20.68%), or four (*n* = 2, 6.89%) biologic disease-modifying antirheumatic drugs (bDMARDs).

**Figure 1:**
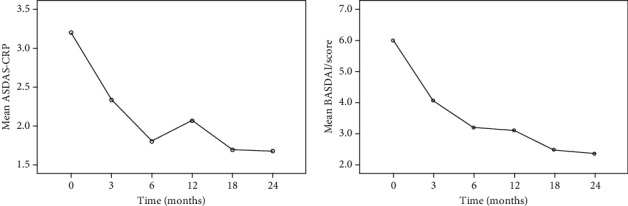
Variations of ASDAS and BASDAI indexes mean values in our cohort over the study period.

The BASDAI score (mean ± SD) was 5.99 ± 1.67 at baseline and 2.35 ± 1.54 at the end of the observation period; the ASDAS-CRP values (mean ± SD) were 3.21 ± 0.9 at the start of treatment and 1.67 ± 0.88 at the end of the observation period. Both BASDAI and ASDAS-CRP showed a statistically significant reduction in the observation period (ANOVA *p* < 0.0001 and *p* < 0.0001, respectively). Post hoc analysis showed significant differences between baseline and every other follow-up visit (*p* < 0.0001).  Figure 1 shows the variations of ASDAS-CRP and BASDAI mean values in our cohort of axSpA patients during the observation time.

The ASDAS-CRP Δ (mean ± SD) between baseline was 1.01 ± 1.03 at the 3-month evaluation and 1.43 ± 1.32 at the six-month follow-up visit.

At the laboratory assessment, CRP significantly decreased in the global observation period (Kruskal-Wallis test: *p* = 0.006), with significant reduction at the post hoc analysis between baseline and both 6-month evaluation (*p* = 0.02) and 24-month visit (*p* = 0.036). No statistically significant variation of ESR emerged between baseline and the follow-up assessments (Kruskal-Wallis test: *p* = 0.270).

No statistical significance was observed in BASDAI and ASDAS-CRP improvement (*p* = 0.482 and *p* = 0.164, respectively) between patients undergoing SCK 150 mg and subjects administered with SCK 300 mg. No significant differences emerged in the BASDAI and ASDAS-CRP variations between biologic-naïve patients and subjects previously failing to TNF-*α* inhibition (*p* = 0.53 and *p* = 0.148, respectively). Similarly, no differences emerged for ESR and CRP variations between dosages administered (*p* = 0.404 and *p* = 0.604, respectively). No significant differences in BASDAI and ASDAS-CRP improvement were observed between psoriatic and non-psoriatic patients (*p* = 0.74 and *p* = 0.58, respectively), as well as between patients diagnosed with radiographic and non-radiographic axSpA (*p* = 0.23 and *p* = 0.90, respectively).

**Figure 2:**
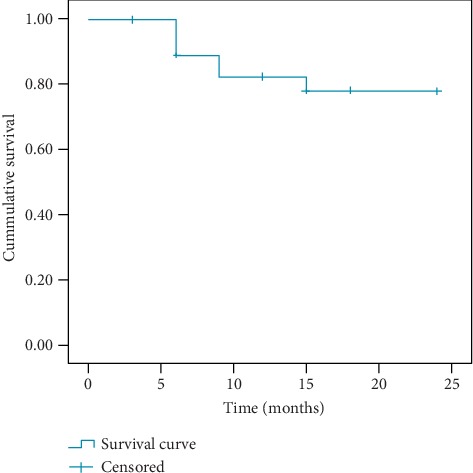
The survival Kaplan-Meier curve of our axSpA cohort treated with SCK.

The global drug retention rate at the end of the study period was 78.2%, without any significant differences between biologic-naïve and anti-TNF-*α*-failure patients (*p* = 0.619) or between subjects administered with different SCK dosages (*p* = 0.614). Similarly, no differences in retention rate emerged between psoriatic and non-psoriatic patients as well as between radiographic and non-radiographic axSpA (*p* = 0.69 and *p* = 0.35, respectively).  Figure 2 shows the Kaplan-Meier survival curves of our cohort.

Concerning safety, no adverse events were reported during the observation time. No tuberculosis reactivation was observed in our cohort, which included 4 patients with latent tuberculosis receiving isoniazid prophylaxis.

## 4. Discussion

In the last few years, management of axSpA has radically evolved with the introduction of biologic cytokine inhibitors like TNF-*α* and IL-17 blockers. Moreover, with the upcoming introduction of the Janus kinase (JAK) inhibitors, there will be a further increase in therapeutic options for managing the most severe cases of axSpA. In this background, our present study intends to provide information about long-term efficacy and tolerability of SCK in axSpA patients.

More specifically, aside from basic efficacy of SCK, we focused on searching for any differences of clinical response between patients treated with different dosages or in different LoBT. Another key aspect we wanted to point out, in consideration of the upcoming increase in treatment options, was indeed the overall drug survival in this real-life context.

About clinical response, SCK induced a significant reduction of ASDAS-CRP and BASDAI parameters over the period of observation and, specifically, with a considerable decrease in the first 3-6 months of treatment. Regarding laboratory assessment, CRP showed a significant reduction over the period of observation, while ESR decrease did not reach a statistical difference between baseline and control visits. These data confirm the prompt efficacy of SCK on axSpA symptoms and systemic inflammation, and also its ability to maintain a therapeutic effect over a considerable amount of time.

In addition, the overall SCK retention rate in our cohort was 78.2%, which we consider a matter of particular interest due to its similarity with data emerging from the randomized controlled trials (RCTs) data in an analogous time of observation [18–20].

Regarding LoBT and dosages employed, our data on the clinical response about treatment with SCK were consistent with our previous short-term experience as well as that of RCTs [18–22], since no differences in clinical response or retention rate were observed between biologic-naïve subjects and in patients formerly exposed to anti-TNF-*α* agents as well as between patients undergoing SCK 150 mg/4 w vs. 300 mg/4 w. Furthermore, no differences in clinical response or drug retention emerged between psoriatic and non-psoriatic patients or between subjects with radiographic vs. non-radiographic axSpA. Although these results were obtained from a fairly small cohort and therefore must be interpreted with caution, our findings again support the adequacy of SCK in the management of axSpA manifestations.

Of note, despite the considerable treatment duration in our cohort, neither adverse events nor infectious diseases were reported during the study period. These data confirm the overall safety profile of SCK, as emerged also by our previous experience [22] and different RCTs, in which the most common adverse events reported were *Candida* infections, upper respiratory tract infections, diarrhea, headache, and nasopharyngitis [27].

We need to recognize the limitations of our study, i.e., the retrospective data collection, the lack of data about extra-articular involvement, the small sample size, and the lack of data about psoriasis clinical response to treatment. However, to the best of our knowledge, this is the first real-life report about SCK long-term effectiveness and drug survival in axSpA patients. In conclusion, SCK has proved to be effective and safe in the management of a real-life heterogeneous cohort of axSpA patients with a remarkable drug retention rate.

## Figures and Tables

**Table 1:** Demographic and clinical data combined with therapeutic features of patients with axSpA enrolled in our study.

Demographic features	Mean ± SD
Age (years)	53.48 ± 9.72
Disease duration (years)	11.28 ± 9.98
Females/males	27/12
Clinical features	(%)
HLA B27 +	6/39 (15.38%)
Psoriasis	19/39 (48.71)%
Radiographic SpA	26/39 (66.66%)
Peripheral involvement	31/39 (79.48%)
GI tract involvement	0/39 (0%)
Ocular involvement	4/39 (10.25%)
PPD test +	4/39 (10.25%)
QFT +	4/39 (10.25%)
Therapeutic features	(%)
Previous anti-TNF failure	29/39 (74.35%)
Use of concomitant GC	11/39 (28.20%)
Use of concomitant DMARDs	14/39 (35.89%)
SCK 150/mg/injection	17/39 (43.58%)
SCK 300/mg/injection	22/39 (56.41%)

GC: glucocorticoids; GI: gastrointestinal; HLA: human leukocyte antigen; PPD: purified protein derivative; QFT: quantiFERON-TB; SCK: secukinumab; SD: standard deviation; SpA: spondyloarthritis; TNF: tumour necrosis factor.

## Data Availability

The data used to support the findings of this study are available from the corresponding author upon request.
